# An Overview of Lung and Breast Cancer Using the National Cancer Database

**DOI:** 10.31557/APJCP.2020.21.1.163

**Published:** 2020

**Authors:** Leigh Deshotels, Gerard Chaaya, Takefumi Komiya

**Affiliations:** 1 *Department of Internal Medicine, *; 2 *Section of Hematology and Medical Oncology, School of Medicine , Tulane University, 1430 Tulane Ave., New Orleans, LA, *; 3 *Parkview Health, Parkview Cancer Institute, 11050 Parkview Circle, Fort Wayne, IN, 46845, U S A. *

**Keywords:** Lung cancer, breast cancer, National Cancer Database, national registry data

## Abstract

**Introduction:**

The National Cancer Database (NCDB) is a clinical oncology database utilized by many researchers and clinicians internationally. We sought to investigate the various trends in data of two of the most common cancers, breast and lung, published using the NCDB.

**Materials and Methods:**

We selected a multitude of pre-determined variables for analysis. We then performed two separate literature searches using an advanced PubMed search builder, and the data was combined to determine each variables’ association with journal impact factor (IF) using both univariate and multivariate analyses.

**Results:**

A total of 191 published studies were identified. We found that a journal IF > 5 was associated with a publication year prior to 2017 (univariate analysis OR 2.68, 95% CI 1.38-5.21, p-value 0.004 and multivariate analysis OR 3.47, 95% CI 1.62-7.42, p-value 0.001) and a sample size > 10,000 (univariate analysis OR 3.27, 95% CI 1.43-7.50, p-value 0.005 and multivariate analysis OR 4.68, 95% CI 1.89-11.6, p-value 0.0008). Variables such as number of authors, region, cancer type, stage, treatment outcome and treatment incidence were not significant for an association with an IF >5.

**Conclusion:**

Based on our data, studies published after 2017 using the NCDB were associated with a lower IF. This could suggest that the quality of the NCDB data may be declining over time, or NCDB is becoming more widely used.

## Introduction

National registry databases are an important component of clinical research as they are heavily relied upon by clinicians and researchers internationally. One of those is the National Cancer Database (NCDB), a joint program of the Commission on Cancer (CoC) of the American College of Surgeons and the American Cancer Society, which uses hospital registry data to track patients with malignant oncologic conditions. (Boffa et al., 2017). It was first established in 1988, and since then, it has incorporated data from more than 34 million historical patient records which covers more than 70% of newly diagnosed cancer cases nationwide (Boffa et al., 2017). This data is used to further oncologic treatment and patient care as well as explore trends in cancer care and serve as quality improvement. 

The NCDB collects data on all types of cancer. Data elements are collected and submitted to the NCDB using a number of web-based data applications developed by the CoC. These hospitals that submit the data must be a designated CoC-accredited hospital. This could limit generalizability as safety-net hospitals are underrepresented, and COC-accredited hospitals only represent about one-third of hospitals nationally (Jagsi et al., 2014). Also, NCDB data is not population-based as is another popular registry database Surveillance, Epidemiology, and End Results Program or SEER (Mohanty and Bilimoria, 2014). NCDB does, however, incorporate various forms of data such as treatment plans, comorbidities, insurance status and overall survival.

We sought to investigate the various trends in data published using the NCDB, and more importantly, the quality of this data as this has only been evaluated in a limited manner. We chose to focus on data surrounding two of the most common types of cancers, breast and lung.

## Materials and Methods

We first selected a number of pre-determined variables that we intended to analyze. We then performed two separate literature searches in March 2019 using an advanced PubMed search builder: one using “breast cancer” and “national cancer database” and another using “lung cancer” and “national cancer database.” Included articles for analysis were those that were published in English and those that used lung and breast cancer cases derived from NCDB.

The following variables were extracted from each article: number of authors (< 5 or > 5), year of publication (< 2017 or > 2017), sample size (< 10,000 or > 10,000), region (US or non-US), cancer type (lung or breast), stage (excluded stage IV or included stage IV), treatment modality incidence (surgery, chemotherapy, radiation therapy) and treatment outcome (survival and mortality). We then searched for each journals’ most recent impact factor (IF) listed on their website. IF was divided into two groups, IF < 5 and IF > 5. All data points were charted in an excel document, and a chi-square analysis was performed looking at each variable and its association with IF. A p-value <0.05 was considered as statistically significant. 

This study was not considered as human subject research that requires Institutional Review Board (IRB) approval.

## Results

There were a total of 191 combined breast and lung cancer studies that met our criteria for analysis. According to Figure I, the number of publications steadily increased over time after 2013 with the exception of an isolated downtrend of lung cancer publications in 2016. The journal with the most publications overall was Annals of Surgical Oncology, and the institution responsible for producing the most publications overall was Yale University School of Medicine in New Haven, Connecticut (Table 1).

A journal impact factor > 5 had a significant association with a publication year prior to 2017 (univariate analysis OR 2.68, 95% CI 1.38-5.21, p-value 0.004 and multivariate analysis OR 3.47, 95% CI 1.62-7.42, p-value 0.001) and a sample size > 10,000 (univariate analysis OR 3.27, 95% CI 1.43-7.50, p-value 0.005 and multivariate analysis OR 4.68, 95% CI 1.89-11.6, p-value 0.0008) (Table 2 and Figure 3). Variables such as number of authors, region, cancer type, stage, treatment outcome and treatment incidence were not significant for an association with an impact factor >5.

**Table 1:** Characteristics of NCDB Studies Published in PubMed

Characteristics	Year of Publication	N	Top Journals	Rank	Name	IF
	<2017	62		1	Lancet Oncology	36.148
	>2017	129		2	J Clin Oncol	26.303
Sample size				3	JAMA Oncol	20.871
	<10,000	61		4	Ann Oncol	13.93
	>10,000	130		5	J Natl Cancer Inst	11.238
No. of authors						
	≤5	52	Top Institutions	Rank	Name	N
	>5	139		1	Yale Univ	36
Region				2	Washington Univ	19
	Non-US	26		3	MD Anderson	18
	US	165		4	U of Chicago	15
Cancer type				5	U of Penn	9
	Lung	109		5	Mayo Clinic	9
	Breast	82				
Stage						
	including IV	40				
	excluding IV	151				
Treatment outcome						
	Yes	141				
	No	50				
Treatment incidence						
	Yes	36				
	No	155				
Impact factor						
	<5	141				
	>5	50				

IF, impact factor; N, number

**Table 2:** Univariate and Multivariate Analyses for Association with Impact Factors Higher than 5.0.

Variable	Univariate analysis			Multivariate analysis		
	OR	95% CI	P-value	OR	95% CI	P-value
Year (<2017 vs. 2017≤)	2.68	1.38-5.21	0.004	3.47	1.62-7.42	0.001
Sample size (10,000< vs. <10,000)	3.27	1.43-7.50	0.005	4.68	1.89-11.6	0.0008
No. of authors (5< vs. ≤5)	1.51	0.70-3.22	0.291	1.56	0.66-3.65	0.306
Region (non-US vs. US)	1.26	0.51-3.11	0.615	2.24	0.78-6.41	0.133
Cancer type (lung vs. breast)	1.73	0.89-3.39	0.108	1.77	0.83-3.78	0.138
Stage (including IV vs. excluding IV)	1.05	0.48-2.30	0.898	1.16	0.45-3.00	0.765
Treatment outcome (Yes vs. No)	2.30	1.00-5.32	0.051	2.38	0.47-12.0	0.292
Treatment incidence (No vs. Yes)	2.62	0.96-7.15	0.061	1.63	0.27-9.75	0.592

OR, odds ratio; CI, confidence interval

**Figure 1:**
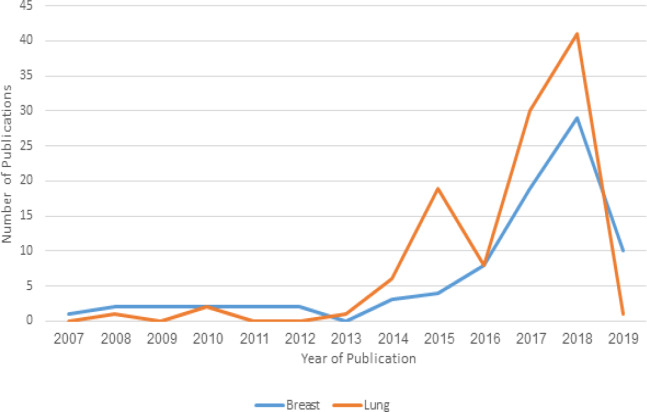
Trends in the Number of Publications Over the Years

**Figure 2:**
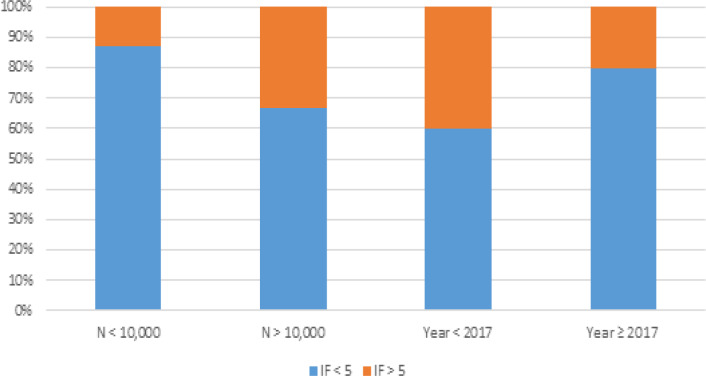
Year and Sample Size and Their Association with Impact Factor

**Figure 3:**
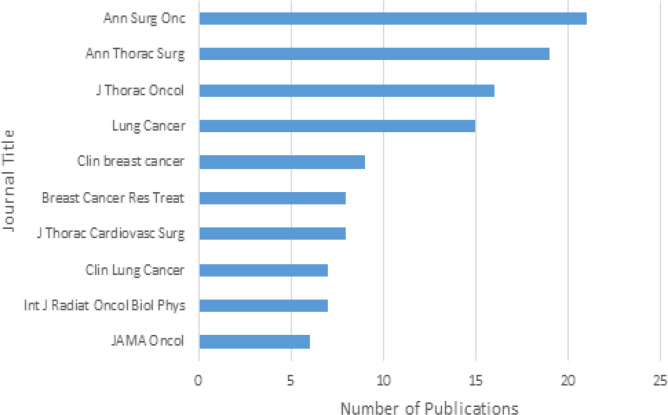
Top Ten Journals with the Most Publications

## Discussion

Based on our data, studies published after 2017 using the NCDB were associated with a lower impact factor. This is despite the fact that the number of publications using NCDB has been increasing over time. This could suggest that either the quality of the NCDB data is declining over time, or the NCDB is becoming more widely used given the increase in publications since 2013. We reported a similar outcome for lung cancer data using the SEER-Medicare database discussed below. We found a significant association between early publication year and high impact factor (Komiya et al., 2018). This was thought to be explained by increased utilization of the SEER database amongst lower impact journals.

We expected that smaller studies were to be underrepresented in journals with an impact factor > 5 given the fact that higher impact factor journals are more likely to publish large data analysis. It, however, may also indicate that the use of the NCDB as a research tool has become more common amongst oncology researchers, and that its impact in the oncology society may be decreasing.

The majority of journals with the highest impact factor were not among the top journals with the most publications as seen when comparing Table I to Figure II. It appears that only JAMA Oncology, which has an impact factor of 20.871, is represented amongst the top ten journals with the most publications. This is despite the fact that these top ten journals represent 61% of the total articles analyzed. This observation was also noted (Su et al., 2018) which analyzed characteristics of all types of cancers published using the NCDB. In their study, around 20% of NCDB papers analyzed by the group were published in journals with IF > 10 (Su et al., 2018). It was expected to see less publications in the highest IF journals, but again, our data is showing a limited acceptance of papers using NCDB data when submitted to the most prominent journals. 

We also observed that there was a decline in the number of lung cancer publications in 2016 followed by a sharp increase in 2017. The theory behind this is unclear. Lung cancer researchers might have become more aware of NCDB around this time. Yale University School of Medicine produced the most number of publications overall. Publications affiliated with the university made up almost 20% of the total publications analyzed. This is followed by Washington University in St. Louis. According to Table I, it appears most of the top academic institutions are utilizing NCDB frequently.

Again, it is important to highlight the distribution and quality of data that comes from large registry databases as they are the cornerstone for oncologic research. Retrospective studies rely heavily on national registry databases, especially the NCDB as it is one of the largest in the nation. As we mentioned earlier, SEER is its main competitor; however, there are major differences between the two. The NCDB allows for cancer surveillance and monitoring outcomes whereas SEER is epidemiological in that it is utilized mainly for measuring cancer incidence (Mohanty and Bilimoria, 2014). However, an additional program, SEER-Medicare, more closely resembles the NCDB in that it provides a link between patient information provided in the Medicare database and SEER database which allows for some monitoring of outcomes (Komiya et al., 2018). One major strength of NCDB compared to SEER is that it allows for quality improvement. Since the NCDB reports cancer outcomes, hospitals are able to use this data to measure their performance compared to the other 1500 CoC-accredited hospitals (Mohanty and Bilimoria, 2014). This is not possible with the SEER database.

We realize the limitation of our analysis. Publications must have been written in English and listed in PubMed. This search method may overlook studies published in non-English or journals not listed in PubMed. Also the cut-off value of IF of 5 was arbitrarily set because its close to a mean number of 4.01. IF has been known to fluctuate over the years, and it is influenced by all the articles and journals in the research field at the time of publication. In addition, our study only analyzed lung and breast cancer publications. Nevertheless, it is widely used and is one of the few tools to judge quality of the journals at this time.

In conclusion, our study found that recent studies using NCDB for lung and breast cancers have a trend of decreasing IF over the last few years. It suggests its becoming a more widely used tool, or its impact in oncology field is deteriorating. As a convenient tool in retrospective oncology research, NCDB will still remain valuable for academic researchers.
